# Implementing a Global Expanded Access Program (EAP) for Infantile-Onset Spinal Muscular Atrophy (Type I): Understanding the Imperative, Impact and Challenges

**DOI:** 10.3233/JND-190387

**Published:** 2019-05-21

**Authors:** Jonathan Yong, Megan Moffett, Sam Lucas

**Affiliations:** Biogen, Cambridge, MA, USA

**Keywords:** Compassionate use, spinal muscular atrophy, expanded access trial, rare disease, antisense oligodeoxyribonucleotides, drug therapy, orphan drug, antisense oligonucleotides, neuromuscular disease

## Abstract

Nusinersen is the first disease-modifying therapy approved for the treatment of spinal muscular atrophy (SMA), a rare genetic disorder characterized by severe progressive muscular atrophy and weakness. An expanded access program (EAP) provides investigational treatment to patients without other treatment options. An EAP providing nusinersen treatment to individuals with the most severe form of SMA, infantile-onset SMA (consistent with SMA Type I), has enrolled over 800 participants as of September 2018, making it one of the largest in rare disease history. The successes, challenges experienced and opportunities for future consideration during the implementation of the nusinersen EAP are discussed.

## BACKGROUND

Spinal muscular atrophy (SMA) is caused by mutations/deletions in the survival motor neuron 1 (*SMN1*) gene, resulting in decreased SMN protein expression and degeneration of spinal cord and brainstem alpha motor neurons [1]. SMA is a rare disease characterized by severe progressive muscular atrophy and weakness, with an incidence of 8.5–10.3 per 100,000 live births [1–4]. SMA disease manifestation is variable [5, 6]. The most severe form is SMA Type I, which is generally diagnosed within 2 weeks to 6 months after birth and requires the most intensive and supportive life-long care [5, 6]. SMA Type I symptoms include severe generalized hypotonia, weakness of the limbs/neck, areflexia, tongue fasciculations, sucking/swallowing problems and abdominal paradoxical breathing or respiratory failure [5, 6]. In the absence of treatment, respiratory support and nutritional interventions, the life expectancy for infants with SMA Type I is < 2 years [6, 7].

Nusinersen is an antisense oligonucleotide drug that modifies splicing of *SMN2* precursor mRNA, resulting in increased full-length SMN protein production [8]. The efficacy and safety of nusinersen have been evaluated in clinical trials in individuals with presymptomatic, infantile-onset and later-onset SMA. Nusinersen demonstrated clinically meaningful efficacy on motor milestone achievement and on survival endpoints and a favorable benefit:risk profile [9–12]. Earlier treatment with nusinersen may maximize treatment benefit [10–12].

Following the positive interim analysis of the ENDEAR trial in infantile-onset SMA (most likely to develop SMA Type I; NCT02193074), the sponsors, Biogen and Ionis Pharmaceuticals, Inc., closed the trial early, and all participants could receive nusinersen in the SHINE open-label extension study enrolling participants who had previously participated in nusinersen investigational studies (NCT02594124) [10]. Nusinersen was the first disease-modifying therapy for the treatment of SMA [13]; thus, demand for this treatment was high. The sponsor launched an expanded access program (EAP) in September 2016 providing nusinersen to eligible individuals with infantile-onset SMA (consistent with SMA Type I) who had not previously participated and were not eligible to participate in nusinersen investigational studies (NCT02865109).

An EAP is a non-promoted, optional program that provides eligible patients with access to an investigational treatment, before regulatory approval and commercial availability [14, 15]. EAPs, also known as Compassionate Use or Named Patient programs, are most commonly offered for rare diseases for which treatment options are unavailable or not tolerated, and typically for severe or life-threatening diseases [15].

Shortly after the ENDEAR trial closed, the EAP was made available to existing nusinersen clinical trial sites across ten countries through a phased rollout. Based on significant need from physicians and patients, the EAP was subsequently broadened to additional treatment sites worldwide where long-term access to nusinersen was anticipated. The decision to restrict the EAP to individuals with SMA Type I [16], as opposed to including other SMA types, was based on the severity and high risk of mortality associated with this type. In each country, the EAP was planned to end when commercialization and reimbursement of nusinersen were achieved. The local country medical teams from the sponsor worked closely with treating physicians to ensure that all participants smoothly transitioned onto commercial drug without treatment interruption.

## ENROLLMENT

**Fig. 1:**
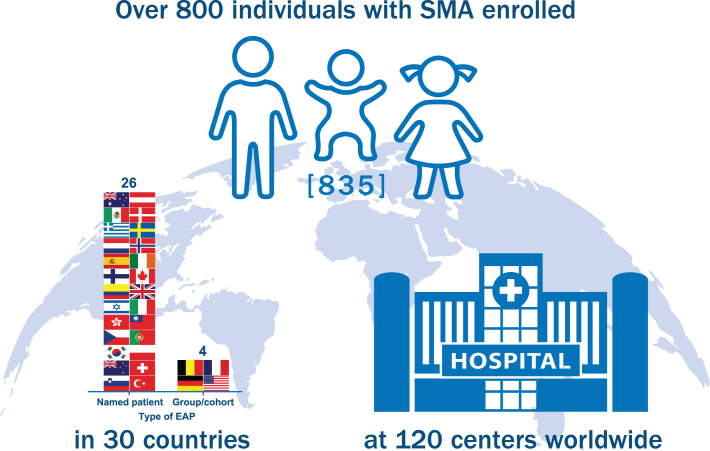
Nusinersen expanded access program (EAP) enrollment. The nusinersen EAP has become one of the largest in rare disease history, enrolling 835 individuals with spinal muscular atrophy (SMA) in 120 centers across 30 countries as of September 20, 2018. Countries utilizing a Named Patient EAP mechanism included Australia, Austria, Canada, Colombia, Czech Republic, Denmark, Finland, Greece, Hong Kong, Ireland, Israel, Italy, Mexico, Netherlands, New Zealand, Norway, Poland, Portugal, Slovenia, South Korea, Spain, Sweden, Switzerland, Taiwan, Turkey, and the United Kingdom. Countries utilizing a Group/Cohort EAP mechanism included Belgium, France, Germany, and the United States. Of note, France initiated EAP participation using a Named Patient program (Nominative temporary authorization for use [ATU]) and later transitioned to a Group/Cohort program (Cohort ATU).

**Fig. 2:**
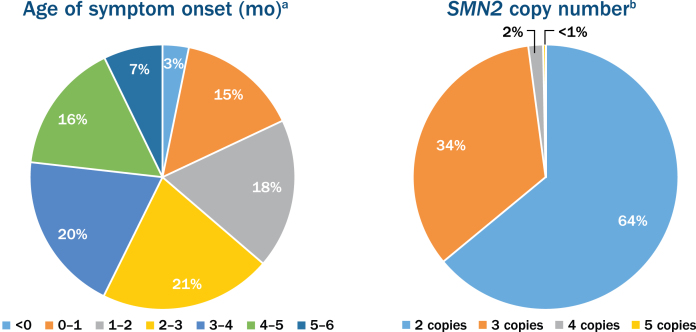
Age of symptom onset and the survival motor neuron 2 (*SMN2*) copy number in participants enrolled in the nusinersen expanded access program. ^a^Out of the 776 participants with age of onset of symptoms ranging from 0 to 6 months. ^b^Out of the 381 participants who reported *SMN2* copy number.

The nusinersen EAP has become one of the largest EAPs developed in rare disease history (Fig. 1). Of the initially enrolled participants, 21.3% were < 2 years old, 75.2% were 2–18 years old and 3.5% were > 18 years old. The overall average age was 4.5 years. Fig. 2 shows additional enrollment statistics.

## IMPACT AND SUCCESSES

Nusinersen treatment significantly improved motor function and extended survival without the need for permanent ventilation in infants with infantile-onset SMA (most likely to develop Type I) [10]. With the implementation of the nusinersen EAP, eligible individuals with SMA Type I were able to access nusinersen treatment up to 3 years before drug reimbursement and commercial launch. Bridging access to therapy was crucial for these participants who, with nutritional and respiratory interventions, still face a mortality rate of approximately 30% at 2 years, with about half of survivors fully dependent on non-invasive ventilation [6].

The nusinersen EAP and clinical study program allowed over 1,150 individuals across a spectrum of SMA types and ages to receive nusinersen before commercial availability, often significantly earlier than the commercial availability of nusinersen in their region.

Despite the scale of the nusinersen EAP, many countries involved and quantity of drug shipments required, the program had a shipment success rate of 99.67% (cold-chain drug deliveries completed on time/within temperature range). The EAP was available in many countries not normally considered for EAPs due to non-existent or complex legislation or uncertain reimbursement prospects for rare disease drugs. For example, the sponsor worked with the Ministry of Health and local patient advocacy groups to develop a compliant framework to facilitate the EAP in Poland despite there being no official legislation to allow for free-of-charge EAPs. The nusinersen EAP had a significant impact on the SMA landscape in Poland, as reimbursement for rare disease drugs historically takes considerable time to achieve following central European Commission approval.

While there was no real-world data collection component to the nusinersen EAP, data from EAP cohorts in Australia, France, Germany and Italy have been published independently by participating physicians. These initial results were some of the earliest real-world data available and reported achievement of motor function unexpected of individuals with SMA Type I, similar to the published ENDEAR study results, as well as clinically meaningful outcomes in a wider range of individuals with infantile-onset SMA [10, 17–20]. SMA registries were also strengthened by the EAP, as EAP physicians often included data collected in SMA disease registries. Real-world data collection during EAPs is an area of expanding interest, with regulators and payers increasingly recognizing the potential value of these data when reviewing new drug applications and determining drug reimbursement.

## CHALLENGES AND RECOMMENDATIONS

Valuable lessons learned from this EAP can be utilized when considering future EAPs. Participation across countries varied widely due to several factors. For example, administrative cost funding proved difficult to obtain for some treatment sites. This was primarily an issue in the United States and occasionally in European countries with publicly funded health care. There were delays in EAP participation, as treatment sites needed time to establish and prepare their institutions. As with many EAPs, because there was previously no treatment for this disease, the contrast between and transition from a palliative and academic paradigm focusing on studying efficacy and safety to an active treatment paradigm necessitated a reconfiguration and/or buildup of institutional and clinical capacity.

Despite these issues, many physicians reported the ability to treat all suitable individuals with SMA Type I in their region through the EAP [20, 21]. An account of EAP implementation in Italy along with advice on forming a national committee for the management and prioritization of participants between treatment centers has been published. This model was considered highly inclusive, collaborative and effective and is encouraged in other regions [21]. Organized and enthusiastic participation from sites helped the nusinersen EAP run more smoothly. Other important factors that aided EAP implementation included regular and consistent communication with centers, having an affiliate presence in participating countries for on-the-ground medical support, and initiating the EAP at clinical trial sites with physicians experienced with nusinersen and the complex nature of SMA.

Patients travelling abroad to access EAP treatment, i.e. medical tourism, was observed on rare occasions and added significant complexities upon EAP closure in several countries. In some cases, following commercial launch of the drug, patients receiving treatment without local medical coverage were ineligible for commercial treatment and had to travel to another country to continue receiving treatment. Based on this, we recommend considering the risk of medical tourism when initiating an EAP and developing appropriate safeguards to ensure there is sustainable long-term access to therapy for enrolled participants.

The nusinersen EAP was generally well received by the SMA community, but earlier engagement with physicians, treatment centers and advocacy groups on criteria, site selection and rollout plans would have been advantageous. The sponsor’s experience strongly advocates for a “very early” engagement approach in EAP planning during Phase 1 studies, including strong bioethical support, as well as broad clinical expert and disease community engagement. The sponsor suggests that strong transparent communication plans and rationales for key decisions be available in parallel with any key potential study program data readouts.

## CONCLUSIONS

Although considerable resources, time and planning are involved in EAP implementation, the potential positive impact of an EAP for those with serious, immediately life-threatening or rapidly debilitating diseases such as SMA is invaluable. Through the collaborative efforts of the sponsor, patient advocacy groups and Ministries of Health, the nusinersen EAP provided eligible participants with nusinersen treatment up to 3 years before local availability. As legislation governing access to investigational therapies evolves, such as recent “right to try” laws in the United States, pharmaceutical companies must think proactively about offering appropriate early access mechanisms for suitable drugs in their development pipelines. The sponsor recommends proceeding with EAP planning readiness at risk, in time for any critical clinical development program juncture, such as pivotal data readout or decision to file.

## CONFLICT OF INTEREST

Jonathan Yong, MD, Megan Moffett and Sam Lucas are full-time employees of and hold stock/stock options in Biogen.
